# Tissue-specific transcriptome profiling of *Drosophila* reveals roles for GATA transcription factors in longevity by dietary restriction

**DOI:** 10.1038/s41514-018-0024-4

**Published:** 2018-04-17

**Authors:** Adam J. Dobson, Xiaoli He, Eric Blanc, Ekin Bolukbasi, Yodit Feseha, Mingyao Yang, Matthew D. W. Piper

**Affiliations:** 1 Institute of Healthy Ageing, Department of Genetics, Evolution and Environment, University College London, Gower Street, London, WC1E 6BT UK; 2 Berlin Institute of Health, Luisenstraße 56, 10117 Berlin, Germany; 3 Present Address: The Gurdon Institute, University of Cambridge, Tennis Court Road, Cambridge, CB2 1QN UK; 4 Present Address: Institute of Animal Genetics and Breeding, Sichuan Agricultural University, Chengdu, Sichuan China; 5 Present Address: School of Biological Sciences, Monash University, Clayton, VIC 3800 Australia

## Abstract

Dietary restriction (DR) extends animal lifespan, but imposes fitness costs. This phenomenon depends on dietary essential amino acids (EAAs) and TOR signalling, which exert systemic effects. However, the roles of specific tissues and cell-autonomous transcriptional regulators in diverse aspects of the DR phenotype are unknown. Manipulating relevant transcription factors (TFs) specifically in lifespan-limiting tissues may separate the lifespan benefits of DR from the early-life fitness costs. Here, we systematically analyse transcription across organs of *Drosophila* subjected to DR or low TOR and predict regulatory TFs. We predict and validate roles for the evolutionarily conserved GATA family of TFs, and identify conservation of this signal in mice. Importantly, restricting knockdown of the GATA TF *srp* to specific fly tissues recapitulated the benefits but not the costs of DR. Together, our data indicate that the GATA TFs mediate effects of dietary amino acids on lifespan, and that by manipulating them in specific tissues it is possible to reap the fitness benefits of EAAs, decoupled from a cost to longevity.

## Author summary

Ageing human populations present a huge societal challenge, providing motivation to find ways to improve health in old age. Dietary restriction (DR), is one way to improve late-life health of animals from worms to mammals, and perhaps humans. This effect was first oberved over 80 years ago, but the underlying mechanism has proven elusive. In this study, gene expression was profiled in diverse tissues of flies subjected to DR, and from these results a role for proteins called GATA transcription factors was predicted. Reducing expression of GATA transcription factors altered the effect of diet on lifespan, and targeting this knockdown to specific tissues reduced side-effects commonly associated with longevity. Therefore this study predicts that targeting GATA transcription factors in specific tissues may promote the benefits, but not costs, of DR.

## Introduction

How can we counter ageing? Answering this question is a major goal, as ever-increasing human lifespans outpace advances in gerontology at great social, personal and financial cost.^1^ Dietary restriction (DR), a mild reduction in nutrient intake without malnutrition, has the evolutionarily conserved capacity to improve lifelong health, but at a cost of reduced biological fitness and vigour in youth.^2^ Despite having been discovered over 80 years ago,^3^ the molecular mechanisms underpinning DR longevity remain elusive. Elucidating these mechanisms could help isolate the benefits of DR from the corollary costs.

The lifespan benefits of DR can be recapitulated by adjusting the relative abundance of nutrients, without restricting the amount of food consumed.^4–6^ In *Drosophila*, the ratio of dietary sugar to yeast modulates lifespan, which is explained by essential amino acids (EAAs) from the yeast.^7^ Importantly, this mechanism is conserved in mice.^8,9^ EAAs up-regulate Target of Rapamycin (TOR) signalling,^10^ and recent evidence indicates that the phenotype of EAA-restricted *Drosophila* is recapitulated by pharmacologically suppressing TOR.^11,12^ Understanding of how TOR curtails lifespan is incomplete, although maintenance of proteome quality likely plays a role.^13–15^ TOR also affects transcription,^16–18^ but to date this output has been relatively poorly studied.

In *Drosophila*, transcriptomic responses to DR have been characterised at the cellular and organismal levels.^18^ Characterising tissue-specific transcription may prove key to understanding the nature of the trade-off between lifespan and fitness imposed by DR. EAAs are unusual nutrients since, by definition, they can be neither synthesised nor stored. Therefore, their dilution in the diet likely changes EAA levels uniformly across tissues. However, this altered signalling may be lifespan-limiting in only a subset of the affected tissues. In this case, longevity may be achieved by reprogramming a DR-like signalling state specifically in those tissues, whilst sparing other tissues of this manipulation would promote their optimal function. Such a manipulation would offer the benefits of an EAA-replete diet, decoupled from its pernicious lifespan-shortening effects. To date, there is evidence from *Drosophila* that benefits of DR are mediated at least in part by the gut^19^ but not the ovary,^20^ suggesting that costs and benefits of dietary change can indeed be partitioned amongst tissues. We have therefore focused on detailing systematically the tissue-specific transcriptional changes induced by DR. We also assess the extent to which DR-dependent transcriptional changes can be explained by TOR signalling, by suppressing its activity in an EAA-replete context, and measuring the degree to which the DR state resembles the low-TOR state. We then predict which transcription factors (TFs) mediate the observed transcriptional changes. We predict and experimentally validate a role for GATA family of TFs in DR longevity. Importantly, we confirm that costs and benefits of DR can be partitioned amongst tissues by targeted TF manipulation.

## Results

This study required precise dietary manipulations. We have developed a semi-defined *Drosophila* diet, which is optimal for early-life egg laying, in which 50% of dietary EAAs are provided as a supplement to yeast-based medium.^11^ Lifespan can be extended by omission of the EAA supplement (i.e., DR), but at a cost of reduced egg laying. Thus, specific EAA dilution provides a precise model to understand how dietary variation influences lifespan, and the associated fecundity cost. Capitalising on this tool and orthology between *Drosophila* and vertebrate organs, we characterised EAAs’ effects on transcriptomes in the brain, fat body (the analogue of the vertebrate liver and adipose), gut, ovary, and thorax (which largely comprises muscle). This enabled us to establish systematically the transcriptional effects of EAA dilution.

### Tissue-specific signatures of DR

**Fig. 1:**
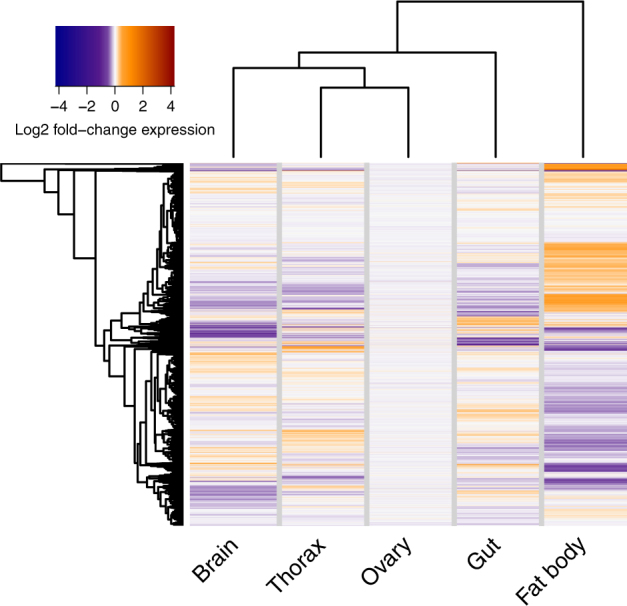
Tissue-specific transcriptomic signatures of DR. The heatmap shows transcriptome-wide log_2_ fold-changes in gene expression induced by DR in each tissue, relative to the EAA-replete control condition. Dendrograms show hierarchical clustering by Euclidian distance

EAAs are required ubiquitously by *Drosophila* tissues, leading us to expect that DR would orchestrate some global changes to gene expression across tissues. We tested for such changes at a transcriptome-wide scale. For each gene in each tissue, a measure of average fold-change in expression upon DR was calculated, and these values were clustered. Contrary to expectation, this analysis did not discriminate any clear clusters (Fig. 1). This result indicated that each tissue under study exhibited a broadly independent transcriptomic response to DR.

To test the tissue-specificity of DR’s effect at a more granular scale, we analysed differential gene expression independently in each tissue. Differentially expressed (DE) genes were detected in all tissues except the ovary (Table 1). Since DR reduces fecundity^11^ and ovary size (unpublished observation), we speculate that DR down-regulates ovariole development, but without altering the transcriptome of each ovariole, with the result that no change was evident at the level of the whole ovary. Evaluating the overlap in sets of DE genes amongst tissues revealed an overwhelmingly tissue-specific effect of DR. No single gene was DE in all tissues. Only five genes (*Cyp9B2*, *tobi*, *CG10910, smp-30*, *CG14661*) were differentially expressed in more than two tissues, and pairwise intersections of DE genes comprised only a small proportion of overall transcriptional change (Supplementary Fig. 1, Supplementary data). Thus, DR had highly tissue-specific effects at the level of gene expression.

**Table 1:** Frequencies of differential gene expression in response to DR per tissue

Tissue	Sign of expression change^a^	N. genes
Brain	Up	60
	Down	144
Fat Body	Up	92
	Down	334
Gut	Up	9
	Down	23
Ovary	Up	0
	Down	0
Thorax	Up	138
	Down	105

^a^Relative to EAA-replete control

Whilst the transcripts affected by DR were highly tissue-specific, a possibility remained that these genes were involved in the same processes, which could lead to equivalent physiological effects of DR amongst tissues. To address this possibility, Gene Ontology (GO) category enrichment was analysed for each tissue’s DE genes. GO analysis revealed that the functional effects of DR were also tissue-specific (Supplementary data). Thus, DR regulates tissue-specific genes, associated with tissue-specific functions.

Overall, our transcriptome analysis indicated that the regulatory effects of DR cannot be understood at the level of the whole organism: instead, gene regulation and function of specific tissues must be studied.

### The DR regulon is enriched in the TOR regulon

Although the transcriptional signatures of DR were highly tissue-specific, these changes were all due to the same upstream stimulus (i.e., EAA dilution). This commonality left open the possibility that similar cellular signalling pathways mediate DR’s effects across tissues. The TOR pathway was a strong target for such a pathway, given its evolutionarily conserved role in signalling EAA availability. In our experimental paradigm, adding rapamycin to the EAA-replete (control) medium promotes phenotypic effects similar to those of DR,^11^ indicating that rapamycin acts downstream of EAAs to promote DR-like signalling. Therefore, we asked whether the transcriptional effects of supplementing the EAA-replete diet with rapamycin were akin to those of DR. These samples were collected in the same experiment, facilitating their direct and quantitative comparison.

**Fig. 2:**
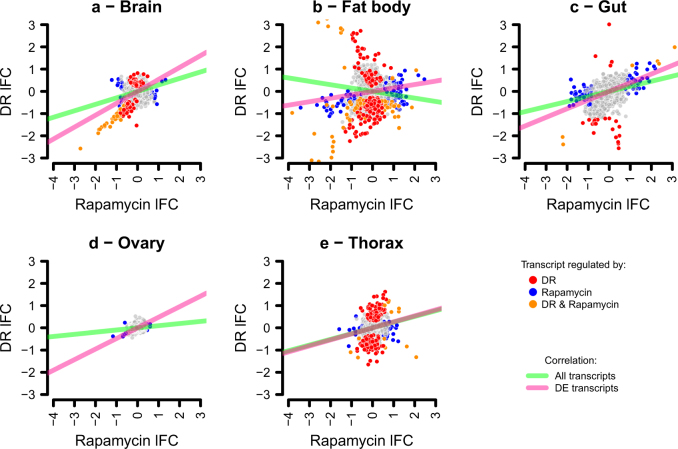
Transcriptional effects of DR are consistent with reduced TOR signalling. Panels show log_2_ fold-changes in expression (lFC) relative to the EAA-replete control in the DR and rapamycin-fed conditions for each tissue assessed (a - brain; b - fat body, c - gut, d - ovary and, e - thorax). Slopes of lines show correlation coefficients (Kendall’s Tau) for all genes in the transcriptome (green), or differentially expressed genes only (pink). All correlations were statistically significant (*p* < 0.05)

To address congruence of DR and rapamycin’s transcriptomic effects, we isolated genes that were DE following rapamycin feeding within each tissue, and examined overlaps within tissues between sets of DR-regulated and rapamycin-regulated genes. Overlaps were examined separately for up-regulated and down-regulated genes, by applying hypergeometric tests. This comparison could not be applied in the ovary, since DR did not cause differential expression there. Rapamycin feeding affected gene expression in all tissues (Supplementary Files). Furthermore, significant overlaps were detected with DR’s transcriptional targets for all tissues, although we note that no genes were up-regulated by both treatments in the brain, and in the fat body the overlap for up-regulated genes was only marginally statistically significant (Table 2). As a second, broad-scale line of testing, within-tissue correlations in changes to gene expression relative to the EAA-replete control were evaluated, both at the level of the whole transcriptome and just DE genes. This analysis revealed correlated effects of DR and rapamycin feeding on DE genes (Fig. 2). Thus, rapamycin feeding not only shared transcriptional targets with DR, but the changes in expression of those targets are quantitatively alike. This pattern was also evident transcriptome-wide, for all tissues but the fat body (Fig. 2). Altogether, these three different analyses show that, for at least one measure per tissue, the transcriptional effects of dietary EAA dilution are consistent with those of reduced TOR signalling, suggesting that TOR signals downstream of DR to regulate gene expression.

**Table 2:** Overlaps within tissues between DR and rapamycin-regulated DE genes

Tissue	Sign expression change^a^	DR	Rapamycin	Overlap	*P*-value^b^
Brain	Up	60	14	0	NA
	Down	144	60	45	2.31e-83
Fat body	Up	92	179	3	0.026
	Down	334	110	43	9.12e-44
Gut	Up	9	52	4	5.76e-11
	Down	23	61	3	2.27e-6
Ovary	Up	0	14	NA	NA
	Down	0	22	NA	NA
Thorax	Up	138	31	8	7.57e-12
	Down	105	15	3	3.20e-6

^a^Relative to EAA-replete control

^b^Hypergeometric test

Whilst DR and rapamycin had overlapping sets of transcriptional targets, this overlap was not complete. To ask whether the functions associated with reduced TOR were consistent with those of DR, we tested the sets of rapamycin-regulated genes for GO enrichment, and compared to the equivalent analysis of DR-regulated genes. Similar GO categories were enriched in the genes regulated by DR and rapamycin (Supplementary Files): For example, in the brain, both DR and rapamycin regulated genes involved in lipid metabolism and cold acclimation, whereas in the fat body both treatments regulated genes involved in antimicrobial defence and vitellogenesis. Thus, rapamycin feeding and DR are predicted to be associated with similar physiological changes.

### Predicting lifespan-regulatory transcription factors

The major goal of our study was to leverage transcriptional data to predict regulators of tissue-autonomous responses to DR. Transcription factors (TFs) were of particular interest, because of their capacity to coordinate multiple transcriptional targets to effect a given physiological programme. We searched the genes that were DE in response to DR for over-representation of motifs known to bind TFs, using a tool that has previously been used to successfully predict TFs that regulate fly phenotype.^21–23^ This analysis revealed a strong enrichment of motifs to bind GATA TFs in all tissues (Supplementary Files). The enrichment was both ubiquitous and highly statistically significant. This association was consistent with prior knowledge of the evolutionarily conserved biology of these TFs: GATA factors play known roles in signalling amino acid availability via TOR in evolutionarily diverse eukaryotes (e.g., yeast^24^ and mosquitos^25^). Furthermore, GATA TFs have known roles in signalling networks governing longevity in *C. elegans*.^26–28^ Thus, the motif enrichment analysis provided a logical result, associating DR-regulated transcripts to TFs with roles in longevity and signalling EAA availability downstream of TOR.

Having established an association between DR and the GATA binding element, we asked whether that signal could be attributed to TOR. We repeated the motif analysis, focusing on the transcriptional targets of rapamycin. Indeed, for all tissues, genes that were DE following rapamycin feeding were also highly enriched in the GATA element. Thus, our results suggested a circuit between circulating EAAs, cellular TOR signalling and the GATA element.

Our results indicated highly tissue-specific transcriptional effects of DR, all of which were associated with TOR/GATA signalling. How could a ubiquitous EAA-TOR-GATA TF circuit achieve tissue-specific transcriptional effects? One possibility is that the identity of the GATA TF TOR signals through depends on the tissue in question. The *Drosophila* genome encodes five GATA factors, and plotting their expression in our transcriptome data revealed that, indeed, each was expressed with its own pattern of tissue specificity (Supplementary Fig. 2). These unique expression profiles may combine with tissue-specific factors to translate systemic signalling into a local gene expression language. Altogether, our results outline a candidate mechanism in which GATA factors act downstream of TOR in diverse tissues to coordinate transcription.

GATA TFs are evolutionarily conserved in vertebrates including mammals, playing important tissue-specific roles in development and disease.^29^ DR and rapamycin promote lifespan of rodents, as well as flies, and the transcriptional effects of both interventions have been established in murine liver by microarray. We asked whether any evidence of GATA signalling could be recovered from these data. Supporting our *Drosophila* data, three of the six GATA TFs encoded by the mouse genome were differentially expressed, either in response to DR or a combined treatment of DR and rapamycin.^30^ Specifically, these microarray data suggested that, relative to a condition of *adlibitum* feeding, *GATAd1*, *GATA1* and *GATA6* were differentially expressed when mice were fed rapamycin and DR food, and that DR alone was sufficient to explain this effect for *GATAd1*. Thus, the association between DR, TOR signalling and GATA TFs appears to be conserved in a vertebrate.

### Functional roles for GATA factors in the DR phenotype

Our results predicted that the tissue-specific activity of GATA factors regulated the effect of DR. We set out to test this prediction for longevity and egg laying, focussing on two GATA TFs in particular: *srp* and *GATAe*. *srp* was of interest because an ortholog of *srp* in *Aedes* mosquitos, *AaGATAa*, links EAAs to oogenesis via TOR and regulation of yolk protein precursors.^25^ This appears to be an evolutionarily conserved function since, in *Drosophila*, it has known roles in regulating oogenesis via yolk proteins in the fat body,^31^ although this function has not yet been connected to EAAs. Given this information and the effect of DR to reduce egg laying, *srp* seemed a likely mediator of responses to DR. The gut is another organ with major roles in the lifespan effect of DR,^19^ so *GATAe* was selected for testing because of its roles in the maintenance of intestinal stem cells,^22^ and the association between their proliferation late in life and longevity.^19^ We tested whether *GATAe* and *srp* in the gut and fat body, respectively, mediated the effect of DR on lifespan and egg laying.

To manipulate *GATAe* and *srp* we expressed RNAi using the well-characterised *TiGS* driver for the gut, and *S*_*1*_*106* for the fat body, which are activated by feeding an inducer, RU_486_. With these tools, we asked whether expressing RNAi against GATAe in the gut or against Srp in the fat body altered the phenotypic effect of DR. To test these responses, we statistically modelled survival using Cox proportional hazards (CPH) analysis, using the main effects of DR and RU_486_, and a DR:RU_486_ interaction term as predictive variables. In this paradigm a significant interaction indicates that the effect of DR is contingent on eating RU_486_, providing a sensitive analysis without a need for pairwise comparisons between experimental conditions.

**Fig. 3:**
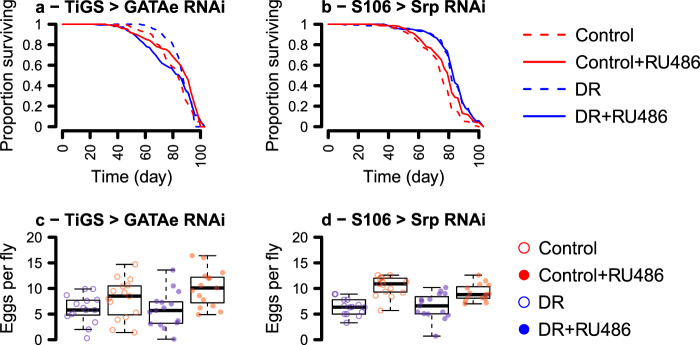
Tissue-restricted knockdown of GATAe and srp interact with dietary EAAs to determine egg laying and lifespan. Survival curves of flies fed EAA-replete or DR media, with/without expression of RNAi against *GATAe* in the midgut (**a**, *TiGS, UAS-GATAe-RNAi*), against *srp* in the fat body (**b**, *S1106, UAS-Srp-RNAi*) Complementary egg laying indices are given in panels **c**–**d** (**c**, *TiGS, UAS-GATAe-RNAi*; **d**, *S1106, UAS-Srp-RNAi*). Control medium was EAA-replete. Survival curves show proportion surviving over time. Box plots show medians, first and third quartiles, and whiskers extend 1.5 × interquartile range, with points showing individual data

In the absence of RU_486_, DR extended lifespan of *TiGS; UAS-GATAe*^*[RNAi]*^ flies (Fig. 3a, Table 3). However, knocking down GATAe altered this effect dramatically. Intestinal *GATAe*^*[RNAi]*^ accelerated the onset of mortality independent of diet but, remarkably, reversed the effect of DR on lifespan: EAAs extended the lifespan of flies expressing intestinal *GATAe*^*[RNAi]*^. Whilst DR extended median lifespan by 6% in the absence of RU_486_, it shortened lifespan by 6% in the presence of RU_486_ (Table 4). CPH analysis detected a significant DR:RU_486_ interaction, confirming that intestinal *GATAe*^*[RNAi]*^ significantly altered DR’s effect on lifespan. Thus, genetic intervention can reverse the sign of DR’s effect on lifespan.

**Table 3:** Cox proportional hazards analysis of DR × GATA RNAi lifespan studies

Genotype	Model term	coef	se(coef)	*z*	Pr( > |z|)
TiGS > GATAe^RNAi^	EAA^a^	−0.3096	0.1305	−2.372	0.0177
	RU^b^	−0.1733	0.13	−1.333	0.18245
	EAA:RU	0.5756	0.1816	3.169	0.00153
S106 > Srp^RNAi^	EAA^a^	0.35421	0.12675	2.794	0.0052
	RU^b^	0.07606	0.12723	0.598	0.5499
	EAA:RU	0.40604	0.1764	2.302	0.0213
DaGS > Srp^RNAi^	EAA^a^	0.6607	0.1247	5.297	1.18E-07
	RU^b^	−0.3105	0.1305	−2.379	0.0173
	EAA:RU	−0.3834	0.1806	−2.123	0.0337

^a^Expressed as effect of adding EAAs, relative to DR medium

^b^Expressed as effect of adding RU_486_, relative to vehicle control

**Table 4:** Median and maximum survival, DR × GATA RNAi lifespan studies

Condition		Lifespan		Sample size	
Genotype	Medium	Median	Maximum	Deaths	Censored
TiGS > GATAe^RNAi^	DR	89	103	126	36
	Control	84	103	127	25
	DR + RU486	86	103	113	41
	Control + RU486	91	103	123	35
S106 > Srp^RNAi^	DR	82	103	128	22
	Control	77	100	142	9
	DR + RU486	84	103	120	31
	Control + RU486	82	103	130	18
DaGS > Srp^RNAi^	DR	77	105	123	29
	Control	72	105	137	9
	DR + RU486	84	107	115	36
	Control + RU486	79	105	123	28

Knocking down Srp also supported a role for GATA TFs in DR longevity. DR extended the lifespan of *S*_*1*_*106/UAS-Srp*^*[RNAi]*^ flies in the absence of RU_486_ (Fig. 3b, Table 3) However, the effect of DR on lifespan was contingent on Srp knockdown, revealed by a significant interaction of RU_486_ and DR in CPH analysis. Fat body *Srp*^*[RNAi]*^ extended median lifespan by 6% on EAA-replete media, but by only 2% on DR media (Table 4). Thus, expressing this RNAi seemed to extend lifespan only when flies were fed the EAA-replete medium, insulating them against the lifespan-shortening effect of the diet. Thus, Srp appears to be required in the fat body for the lifespan-shortening effect of dietary EAAs. Consistent with the study of intestinal GATAe, this result suggests a role for GATA TFs in DR longevity.

An ideal anti-ageing intervention should be effective without biological costs in early life. Reduced egg laying is a well-documented fitness cost of DR in flies. Having shown that the benefits of DR were contingent on GATA TFs, we tested whether they also mediated this cost. We counted eggs laid in early life (day 8 of adulthood) by the same flies that were assayed for survival, and analysed egg laying by fitting the same terms as were fitted for survival in an ANOVA model. Consistent with expectation, in the absence of RU_486_, DR reduced egg laying for both *S*_*1*_*106/UAS-Srp*^*[RNAi]*^ and *TiGS; UAS-GATAe*^*[RNAi]*^ flies. However, neither *Srp*^*[RNAi]*^ nor *GATAe*^*[RNAi]*^ reduced egg laying, nor interacted with the effect of DR on egg laying (Fig. 3c,d, Table 5). Thus, the roles of these GATA TFs in the gut and fat body on DR longevity appears to be independent of DR’s fecundity effects.

**Table 5:** ANOVA of fecundity of flies expressing RNAi against GATA factors

Genotype	Model term	Df	Sum Sq	Mean Sq	*F* value	Pr( > F)
TiGS > GATAe^RNAi^	EAA^a^	1	2.5697	2.56968	9.0973	0.003845
	RU^b^	1	0.1918	0.19179	0.679	0.413429
	EAA:RU	1	0.4408	0.44077	1.5604	0.216796
	Residuals	56	15.8181	0.28247		
S106 > Srp^RNAi^	EAA^a^	1	2.4628	2.46276	29.873	1.15E-06
	RU^b^	1	0.0821	0.08212	0.9961	0.3226
	EAA:RU	1	0.027	0.02702	0.3278	0.5693
	Residuals	55	4.5343	0.08244		
DaGS > Srp^RNAi^	EAA	1	1.9033	1.9033	19.36	5.14E-05
	RU	1	18.8789	18.8789	192.034	< 2.20E-16
	EAA:RU	1	0.0938	0.0938	0.9541	0.333
	Residuals	54	5.3088	0.0983		

^a^Expressed as effect of adding EAAs, relative to DR medium

^b^Expressed as effect of adding RU_486_, relative to vehicle control

**Fig. 4:**
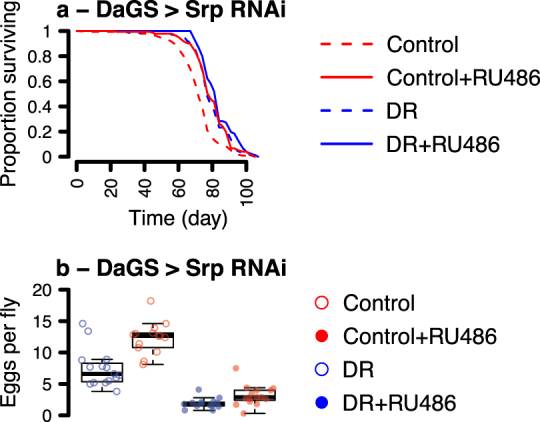
Systemic srp knockdown recapitulates benefits of DR at enhanced cost. Survival curves of flies fed EAA-replete or DR media, with/without expression of RNAi against *srp* under control of *daughterless* GeneSwitch (**a**), with complementary egg laying indices (**b**). Control medium was EAA-replete. Survival curves show proportion surviving over time. Box plots show medians, first and third quartiles, and whiskers extend 1.5 × interquartile range, with points showing individual data

The effect of GATAe and Srp on lifespan but not reproduction indicated that DR’s influence on these life history traits are not obligately coupled by signalling. We hypothesised that this could be explained either by the effect of DR on egg laying and lifespan being mediated by the same mechanism in distinct tissues, and, additionally or alternatively, by distinct mechanisms in the same tissues. *Srp* provided a system to test these models, since our results showed that its expression in the fat body is required for the full effect of DR on longevity but not fecundity; because it is expressed in tissues other than the fat body (Supplementary Fig. 2); and because it is a known regulator of signals that promote oogenesis.^31^ Therefore, we ubiquitously expressed the same *UAS-Srp*^*[RNAi]*^ construct as previously, under the control of the *Daughterless-GeneSwitch (DaGS)* driver. We expected that this systemic manipulation would interact with DR to determine lifespan as previously, but with an additional fecundity effect. Such an effect would indicate a role in reproduction in tissues other than the fat body. Consistent with previous results, ubiquitous *Srp*^*[RNAi]*^ expression altered the effect of DR on lifespan, evidenced by a significant diet:RU_486_ interaction term in CPH survival analysis (Fig. 4a, Tables 3–4). Thus, the effect of knocking down Srp in the fat body had the same qualitative effect as ubiquitous knockdown. We then examined the effect of ubiquitous *Srp*^*[RNAi]*^ expression on egg laying: An interaction between ubiquitous *Srp*^*[RNAi]*^ and DR would suggest a direct role for Srp in mediating diet’s fecundity effects, whilst additive effects would indicate parallel roles. Strikingly, ubiquitous *Srp*^*[RNAi]*^ expression reduced fecundity by an order of magnitude (Fig. 4b). This effect confirmed a role for Srp in egg laying and indicated a strong biological cost of this intervention, in contrast to the fat body-restricted knockdown. However, there was no interaction between the effect of DR and the effect of *Srp*^*[RNAi*]^ (Table 5), consistent with Srp and dietary EAAs affecting fecundity via parallel mechanisms. Thus, the effect on longevity of knocking down Srp appears to be spatially discrete from its fecundity effect, if limited to the fat body. Systemically, its fecundity effect appears to be via a diet-independent mechanism. Thus, whilst all three of the GATA knockdowns in this study interacted with the effect of diet to determine lifespan, none of them interacted with diet to determine fecundity.

## Discussion

DR improves lifelong health in a range of organisms, from yeast to primates. A growing body of evidence shows the particular importance of dietary nutrient balance for ageing, particularly lowered protein:carbohydrate ratio,^32^ which implicates amino-acid sensitive TOR signalling. A likely role of TOR was recently underlined in *Drosophila*, by the demonstration that active TOR is required for EAA enrichment to shorten lifespan.^11^ In the present study we have confirmed that the transcriptional effects of DR are consistent with reduced TOR signalling. Moreover, our results now identify a third level of regulation, implicating the GATA family of TFs as mediators of dietary EAAs’ lifespan effects. The evolutionarily conserved roles of this family of TFs in signalling EAA availability, TOR signalling, oogenesis and tissue-specific gene regulation, in addition to their regulation by DR and rapamycin in murine liver, make them potential mechanisms of longevity assurance worthy of further investigation. Roles of GATA TFs in worm longevity have been established.^26–28^ Mice are also long-lived when fed a restricted diet or rapamycin, and the finding that GATA TFs are differentially regulated in mouse liver,^30^ reveals evolutionary conservation and warrants study of their role in mouse longevity.

The major motivation to understand how DR affects physiology is to identify means to recapitulate its benefits in later life without associated biological costs, not to mention costs of an ascetic lifestyle. Our results show that GATA TFs interact with diet to determine lifespan, but not egg laying–the major fitness cost of DR in *Drosophila*. Furthermore, whilst ubiquitous expression of *Srp*^*[RNAi]*^ did affect egg laying, there was no interaction with diet: in fact, none of the GATA TF knockdowns altered the effect of diet on egg laying. Furthermore, restricting the *Srp*^*[RNAi]*^ to the fat body made the flies long-lived without any effect on fecundity. Thus, GATA TFs are candidates to relax tradeoffs between life-history traits relevant to ageing, particularly when targeted specifically to lifespan-relevant tissues, allowing the benefits of feeding to repletion whilst sparing costs to lifespan.

An important outcome of our analysis is the highly independent transcriptional response of each tissue to DR. We hypothesised that transcriptional programmes under tissue-specific control would be sufficient for longevity benefits of DR, by disconnecting lifespan-limiting pathologies and processes from the homoeostatic regulation of other tissues. Importantly, the experiments involving *srp* knock-down demonstrate that benefits of DR can be reaped by targeting transcription factors in specific organs, making flies constitutively long-lived even when eating an EAA-replete diet. Whilst these results could possibly result from stronger expression of the *DaGS* driver than the *S*_*1*_*106* driver, this seems an unlikely explanation given that both constructs imparted a similar lifespan phenotype, but vastly different egg laying phenotypes. Curiously, *GATAe* knockdown in the gut changed the sign of the effect of DR on lifespan, suggesting either a different optimal dietary balance in these flies, or that elevated EAAs rescued the pathological effects of gut-specific GATAe knockdown. These experiments implicate GATA factors as mechanistic links between diet, tissue-specific gene regulation and lifespan.

The putative connections between lifespan, diet and GATA TFs are entirely consistent with the evolutionarily conserved functions of these proteins. The GATA TFs are an ancient family, with well-characterised roles in development and nutrient signalling. In multicellular differentiated organisms, GATA TFs are required in the development of multiple tissue types, which in *Drosophila* includes the heart,^33^ fat body^34^ and gut.^35^ However, their functions in adulthood are poorly characterised. *GATAe* plays a role in midgut homoeostasis in adult *Drosophila*, but it is unclear where it integrates into broader midgut signalling networks.^36,37^ One of the better-described roles for GATA factors in adult animals is in nutrient regulation of oogenesis in mosquitos.^25,38–40^ In *Aedes aegypti*, oogenesis requires a blood meal, which contains the mosquito’s only source of protein. Before feeding, egg production is suppressed partly by a GATA TF repressing expression of the major yolk precursor protein, Vg, in the fat body. After feeding, TOR enhances expression of the transcriptional activator *AaGATAa*, which de-represses *Vg* expression. This circuit appears to be at least partially conserved, since *srp* regulates yolk protein expression in *Drosophila*,^31^ and our results show that its ubiquitous knockdown strongly attenuates egg laying, although the absence of an interaction with diet indicates a mechanism independent of EAAs. Evidence from yeast suggests that the role of GATA factors in regulating nitrogen metabolism is basal in Eukaryotes. In *Saccharomyces cerevisiae*, selective amino acid catabolism is controlled by a circuit known as Nitrogen Catabolite Repression: when nitrogen availability supports only poor growth, TOR-dependent nuclear localisation of a GATA TF activates expression of genes involved in the transport and metabolism of less-preferred nitrogen sources.^24^ Together, these data highlight conserved connections between protein nutrition, growth, reproduction, TOR and transcriptional regulation by GATA TFs; and we have now connected GATA TFs to longevity by DR. These roles are entirely consistent with evolutionary theories that ageing is a consequence of deleterious pleiotropy with mechanisms under selection in early life.^41^

This study reveals tissue-specific patterns of transcriptional regulation in response to the longevity-promoting restriction of EAAs. The transcriptional effects of EAAs are associated with TOR signalling and motifs to bind GATA TFs, and the tissue-specific activity of GATA factors appears to dictate the effect of diet on phenotype. Importantly, these experiments also suggest that the costs and benefits of dietary variation may be mediated by different tissues, and therefore that benefits may be reaped without fitness tradeoffs by tissue-specific genetic interventions. The evolutionary conservation of GATA factors, of their connection to regulating amino acid metabolism, and of the capacity of DR to mediate lifespan extension, suggests that GATA factors may be relevant to DR’s anti-ageing effect in a broad range of animals.

## Materials and methods

### Diets

Diets were prepared according to ref. ^11^ The base (1SY) medium contained 100 g/l autolysed yeast (MP Biomedicals, OH, USA), 50 g/l sucrose (Tate & Lyle, London, UK), 15 g/l agar (Sigma-Aldrich, Dorset, UK), 30 ml/l nipagin (Chemlink Specialities, Dorset, UK), and 3 ml/l propionic acid (Sigma-Aldrich, Dorset, UK). The EAA-replete control medium comprised DR food with an EAA supplement dissolved in pH 4.5 water (final concentrations in fly media: L-arginine 0.43 g/l, L-histidine 0.21 g/l, L-isoleucine 0.34 g/l, L-leucine 0.48 g/l, L-lysine 0.52 g/l, L-methionine 0.1 g/l, L-phenylalanine 0.26 g/l, L-threonine 0.37 g/l, L-tryptophan 0.09 g/l, L-valine 0.4 g/l: all suppled by Sigma). EAA + rapamycin food consisted of EAA-replete medium with a rapamycin supplement (LC laboratories, MA, USA) dissolved in ethanol, to a final concentration of 200 µM in the diet. For RNAi experiments, RU_486_ (Sigma M8046) dissolved in ethanol was added to 1SY or EAA food to a final concentration of 200 µM.

### Fly culture

Outbred wild-type Dahomey flies bearing the endosymbiont *Wolbachia* were cultured on a 12:12 light cycle at 25 °C and 60% humidity, on 1SY medium. For RNAi experiments, the *TiGS*, *S*_*1*_*106* and *DaGS* drivers, and RNAi constructs (*UAS-srp*^*[RNAi]*^: Vienna Stock Center #35578; UAS-GATAe^TRiP^: Bloomington Stock Center #33748) were backcrossed into Dahomey flies bearing the *w1118* mutation for at least six generations. UAS-GATAe^TRiP^ was backcrossed into this background by genotyping individual flies by PCR of the vector. *S*_*1*_*106* drives in the fat body and anterior midgut, however the effect of gut srp^[RNAi]^ can be excluded owing to the absence of *srp* expression in the gut (Supplementary Files). All flies were maintained at large population sizes to maintain outbred genetic diversity. For all experiments, eggs were collected following an 18 h lay on grape juice agar, added to bottles of 1SY at a standardised density and cultured to adulthood. Newly emerged experimental females were mated on fresh food for 48 h, lightly CO_2_ anaesthetised, then allocated to experimental diets without males (10 females/vial). For egg laying and lifespan experiments, survival was scored and flies transferred to new media at least three times per week. Egg laying was scored on day 8 after eclosion, after 18 h egg laying. Phenotyping experiments were set up at different times, one per experimental genotype, thus multiple experiments validate the role of GATA TFs.

### RNA sequencing

Tissues were dissected in ice-cold RNAlater solution, 6–10 h into the flies’ light cycle, and frozen at −80 °C. RNA was extracted using the QIAGEN total RNA isolation kit and quantified on an Agilent 2100 bioanalyser. Sequencing was performed by the high throughput genomics services center at the Huntsman Cancer Institute (University of Utah). Sample concentration and purity of RNA was measured on a NanoDrop spectrophotometer, and RNA integrity was assessed on an Agilent 2200 TapeStation. Illumina TruSeq libraries were prepared from this RNA with the Illumina TruSeq Stranded mRNA Sample Prep kit and sequenced on an Illumina HiSeq2000 101 v3 platform using paired-end sequencing.

### Data analysis

Reads were aligned to *D. melanogaster* genome v6.19 using HISAT2 v2.1.0 and enumerated using featureCounts v1.6.0. Unmapped reads were discarded. Enumerated reads were analysed in R and bioconductor (3.3.1). Two gut samples from the DR condition were found to be internally inconsistent with other gut samples, and also with gut transcriptomes from external experiments. These samples were therefore excluded from further analysis. Fold-changes in expression were calculated by DESeq2 (1.18.1). Differential expression and fold-changes in expression were determined for each tissue by DESeq2, by fitting a model of the form

$$y\sim diet + replicate$$

where *y* represented read counts of transcript_i_, *replicate* coded for biological replicate with a three-level factor, and *diet* coded for whether the flies were fed control medium, DR medium, or rapamycin-supplemented medium. Contrasts were then applied between the control versus DR condition, and control versus rapamycin-fed condition. *P*-values were corrected using independent hypothesis weighting, with base mean of normalised counts as a covariate. Differential expression was considered statistially significant when corrected *P*-values were ≤ 0.01. Motif enrichment amongst differentially expressed genes was analysed using *i-Cis target*.^42^ Heatmaps were clustered by Euclidian distance metrics and plotted using the *heatmap.2* function from the gplots library, either on fold-changes calculated by DESeq2, or median of variance-stabilised GATA TF expression. Intersections between DE gene sets were visualised using the upset library.^43^

Fly survival and egg laying data were analysed in R (v3.1.1). Survival data were analysed using the *coxph* function from the survival library. Egg laying data were normalised to number of flies per vial and log-transformed, and analysed by fitting a linear model (ANOVA) using the *lm* function. Both survival and egg laying data were analysed with a model of the form

$$y\sim RU + EAAs + RU486:EAAs$$

where *y* coded for survival or egg laying index, *RU* and *EAAs* for the presence of RU_486_ and EAAs, respectively, and the interaction of the two.

### Code availability

All code is available on request.

### Data availability statement

Raw RNAseq data have been deposited with ArrayExpress, Accession E-MTAB-6584. All processed RNAseq data used in Figs. 1 and 2 and phenotypic data used in Figs. 3 and 4 are available in Supplementary Files.

## Electronic supplementary material

Supplementary Figure 1

Supplementary Figure 2

Supplementary data

## Electronic supplementary material

**Supplementary information** accompanies the paper on the *npj Aging and Mechanisms of Disease* website (10.1038/s41514-018-0024-4).
