# Supplementary material for: Tissue-specific transcriptome profiling of Drosophila reveals roles for GATA transcription factors in longevity by dietary restriction
Source: NPJ Aging Mech Dis. 2018 Apr 17;4:5. doi: 10.1038/s41514-018-0024-4 (PMC5904217; doi:10.1038/s41514-018-0024-4)
Supplement: Supplementary file 1 — Supplementary Figure 1 [file 41514_2018_24_MOESM1_ESM.pdf]

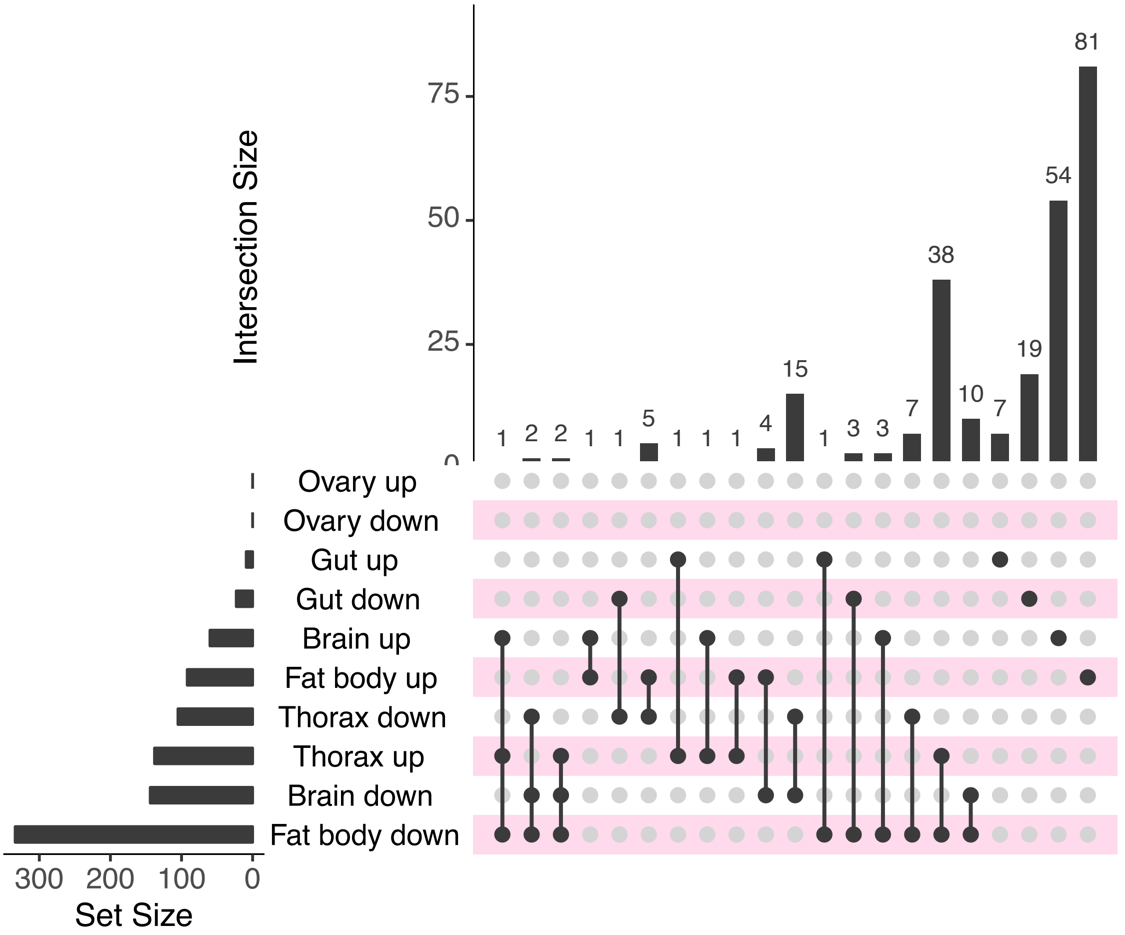


**Supplementary Figure S1. Intersections of DE gene sets amongst tissues.**

Each row of the matrix represents the sets of genes that were DE in response to DR in each tissue. Each column represents the intersection between those sets, visualised by points connecting rows of the matrix. A single point is plotted for genes which are unique to one set. For example, of the five genes upregulated by DR in the gut, four were unique to that set, and one was also down-regulated in the fat body.
