# Supplementary material for: Tissue-specific transcriptome profiling of Drosophila reveals roles for GATA transcription factors in longevity by dietary restriction
Source: NPJ Aging Mech Dis. 2018 Apr 17;4:5. doi: 10.1038/s41514-018-0024-4 (PMC5904217; doi:10.1038/s41514-018-0024-4)
Supplement: Supplementary file 2 — Supplementary Figure 2 [file 41514_2018_24_MOESM2_ESM.pdf]

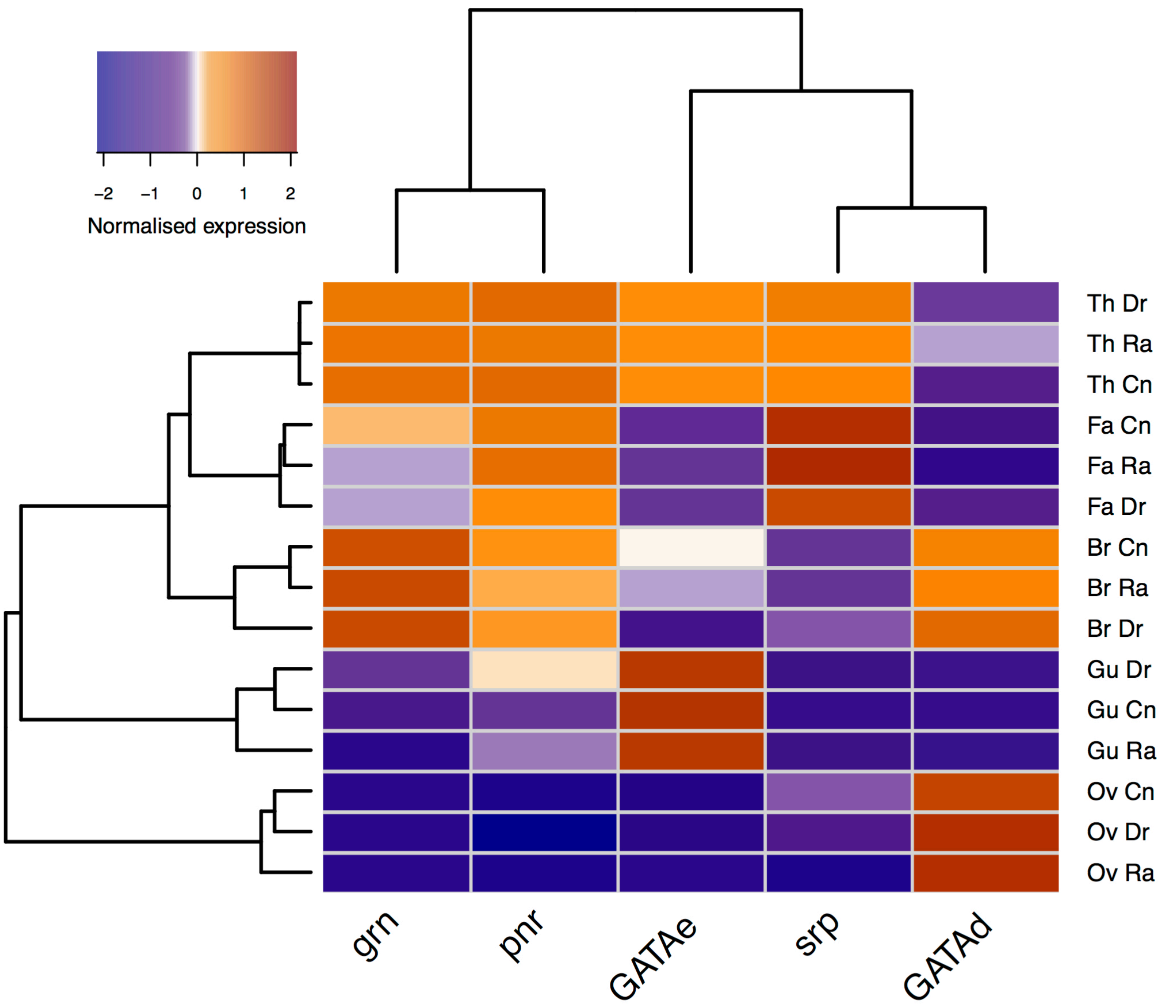

Supplementary Figure S2. GATA TFs are expressed by a tissue-specific pattern. For each of the five *Drosophila* GATA TFs, median expression per tissue and experimental condition is plotted, of variance-stablised read counts. Dendrograms represent heirarchical clustering by a Euclidian distance metric. Row labels indicate tissue (Br=Brain, Fa=Fat body, Gu=Gut, Ov=Ovary, Th=Thorax) and diet (Dr=DR, Ra=Rapamycin, Cn=Control). Values are column-scaled Z-scores of medians of varianced-stabilised reads.
